# A unique case of multiple calvarial hemangiomas with one large symplastic hemangioma

**DOI:** 10.1186/s12883-021-02053-7

**Published:** 2021-01-19

**Authors:** Najwa Abdalkabeer A. Bantan, Ahmed H. Abouissa, Muhammad Saeed, Mustafa Hassan Alwalily, Kamal Bakour Balkhoyour, Khalid Mohammad Ashour, Amal Ali Hassan, Afnan Hisham Falemban, Mohiuddin M. Taher

**Affiliations:** 1 Department of Radiology, Al-Noor Specialist Hospital, Makkah, Saudi Arabia; 2 Department of Neurosurgery, Al-Noor Specialist Hospital, Makkah, Saudi Arabia; 3 Department of Laboratory Medicine, Division of Histopathology, Al-Noor Specialist Hospital, Makkah, Saudi Arabia; 4 Faculty of Medicine, Department of Pathology, Al Azhar University, Cairo, Egypt; 5 Department of Medical Genetics, Umm-Al-Qura University, Makkah, Saudi Arabia; 6 Science and Technology Unit, Umm-Al-Qura University, Makkah, Saudi Arabia

**Keywords:** Calvarial hemangioma, Symplastic hemangioma, Dural sinus, Preoperative embolization, Surgical resection, Saudi Arabia

## Abstract

**Background:**

Symplastic hemangioma is a benign superficial abnormal buildup of blood vessels, with morphological features which can mimic a pseudo malignancy. A few cases have been reported in the literature. We report here, a unique case of calvarial symplastic hemangioma, which is the first case in the calvarial region.

**Case presentation:**

A 29-year-old male patient, with a left occipital calvarial mass since childhood, that gradually increased in size with age, was associated with recurrent epileptic fits controlled by Levetiracetam (Keppra), with no history of trauma. He presented to the emergency room with a recent headache, vomiting, frequent epileptic fits and a decrease in the level of consciousness 1 day prior to admission. A CT scan showed three diploic, expansile, variable sized lytic lesions with a sunburst appearance; two that were biparietal, and one that was left occipital, which were all suggestive of calvarial hemangiomas. However, the large intracranial soft tissue content, within the hemorrhage of the occipital lesion was concerning. The patient had refused surgery over the years; however, after the last severe presentation, he finally agreed to treatment.

The two adjacent, left parietal and occipital lesions were treated satisfactorily using preoperative embolization, surgical resection, and cranioplasty. Histopathology revealed cavernous hemangiomas, in addition to symplastic hemangioma (pseudo malignancy features) on top at the occipital lesion. The right parietal lesion was not within the surgical field; therefore, it was left untouched for follow-up.

**Conclusions:**

Histopathology and radiology examinations confirmed the diagnosis as symplastic hemangioma, on top of a pre-existing cavernous hemangioma. To the best of our knowledge, this is the first case of a calvarial symplastic hemangioma, which we report here.

## Background

Intraosseous hemangioma is a rare bone neoplasm and accounts for < 1.0% of all bone tumors. They are usually found in the vertebral column and are rarely found in the calvarium [1, 2]. Calvarial hemangiomas are benign vascular malformations that account for approximately 0.2% of all bone tumors [3]. Calvarial hemangiomas or cavernomas of the skull are termed intradiploic cavernomas, as they predominantly grow within the diploic space [4]. Approximately 13% of calvarial lesions are hemangiomas, they are slow-growing, and benign. The majority of cases have been reported to contain a single lesion; however, the incidence of multifocal hemangiomas of the skull appears to be extremely rare [5–8]. They are usually asymptomatic, but may become symptomatic if they are large enough, with a compressing effect.

Symplastic hemangiomas have been described more recently, and the main features are degenerative pleomorphism, with atypia in the vascular smooth muscle and stromal interstitial cells in a pre-existing vascular lesion [9, 10]. In surgical pathology, the word ‘symplastic’ was used to describe the bizarre and multinucleated cell morphology, in the absence of other histological criteria of malignancy [11]. Symplastic hemangioma is also a benign superficial, abnormal buildup of blood vessels, however its morphological features resemble with a malignant tumor. The distinctive lack of endothelial nuclear atypia allows it to be distinguished histologically from malignant vascular tumors. The symplastic type of hemangiomas has not been reported in the calvarial part of the skull so far. We present a case of multiple calvarial hemangiomas, with one large left occipital mass since childhood, which was rapidly growing and extending intracranially with a mass effect. Its final pathology was unexpectedly symplastic hemangioma, on pre-existing vascular lesions.

## Case presentation

A 29-year-old male patient, with a left occipital calvarial palpable, hard, non-tender mass since childhood, that gradually increased in size with age, was associated with recurrent epileptic fits controlled by Levetiracetam, with no history of trauma. He presented in the emergency room (ER) with recent headache, vomiting, frequent epileptic fits, and a decrease in the level of consciousness 1 day prior to admission. He was vitally stable (GCS15/15) and afebrile, was able to move all of his limbs actively and was neurologically intact. Computed tomography (CT) of the brain revealed a left occipital and parietal hemangioma and he was admitted under the care of the neurosurgery team to be operated on.

### Radiographic features

**Fig. 1:**
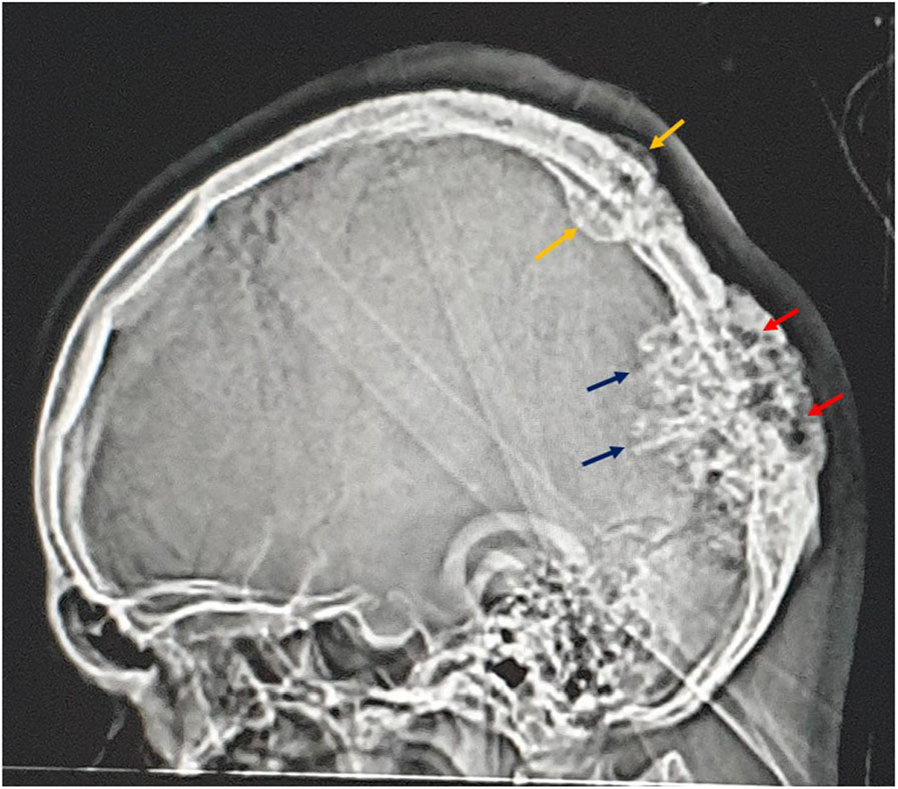
Scout view of preoperative CT scan showing parietal (yellow arrow) and occipital bony lesions. The occipital lesion was larger with internal trabeculations (red arrows), and a major intracranial part with spiculated margins (blue arrows)

**Fig. 2:**
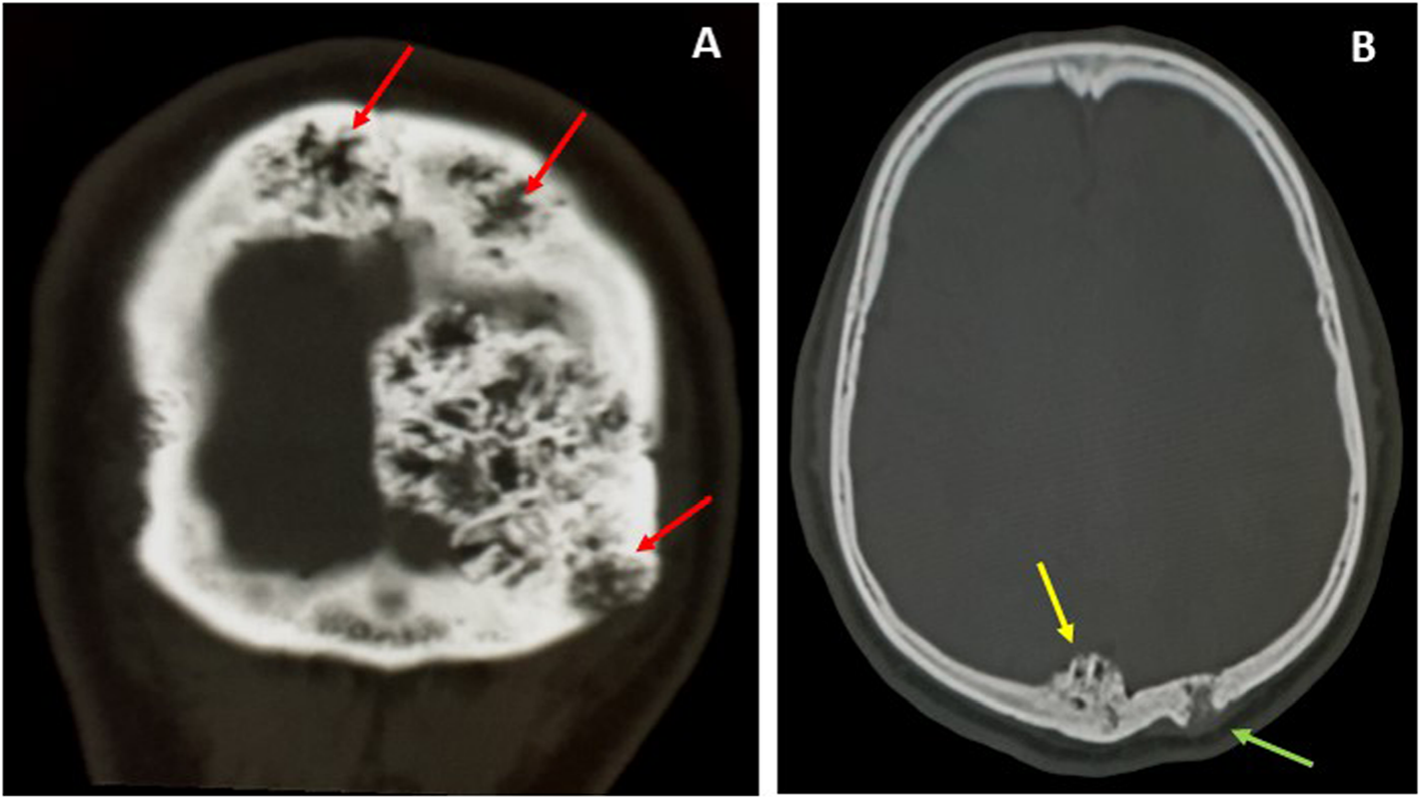
Computed tomography scan bone window coronal image showed 3 variables sized, rounded, diploic, expansile, lytic lesions with sunburst appearance (Red arrows), two biparietal, and left occipital (**a**). CT scan bone window axial image showed biparietal diploic lesions (**b**). The right one without wall erosion (Yellow arrow) while the left one with external wall erosion and subgaleal soft tissue (Green arrow)

**Fig. 3:**
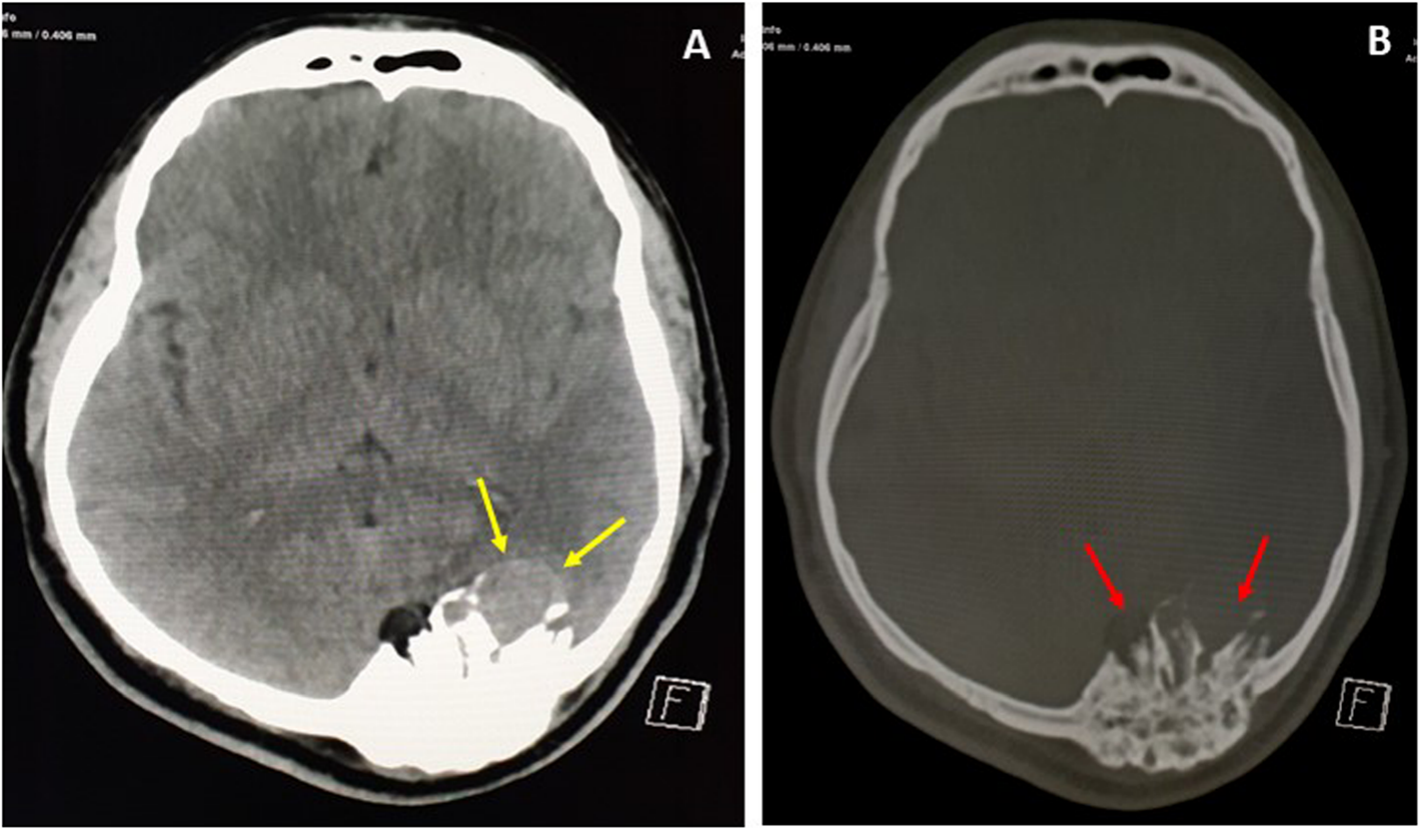
Non-enhanced computed tomography (NECT) scan of brain (**a**); and bone window (**b**) showing large left occipital, expansile, lytic lesion with sunburst (Red arrows) appearance, and intracranial extension with soft tissue (Yellow arrows) component

A skull X-ray was not performed; however, the scout view of the preoperative CT scan showed parietal and occipital expansile lytic bony lesions, involving the inner and outer tables. The occipital lesion was larger, with internal trabeculations and a major intracranial part with spiculated margin (Fig.1). The CT scan showed three variable-sized, rounded, diploic, expansile, lytic lesions, with a sunburst appearance, thin walls and a thick trabecular appearance from the center (Fig.2a). There were two lesions at the bilateral parasagittal posterior parietal bones, measuring 20 and 12 mm in diameter, for the right and left, respectively, with the left one showing external wall erosion and mild subgaleal soft tissue (Fig.2b). In addition to the one large left parasagittal lesion, there was a left occipital bone lesion crossing the midline, measuring around 5 cm in diameter, with focal external wall erosion, internal wall defect and large epidural soft tissue. The cystic mass was compressing the adjacent occipital lobe and the occipital horn of the left lateral ventricle, and deviating the splenium of the corpus callosum to the right (Fig.3a). The soft tissue component was mildly hyperdense relative to the gray matter (Fig.3b).

**Fig. 4:**
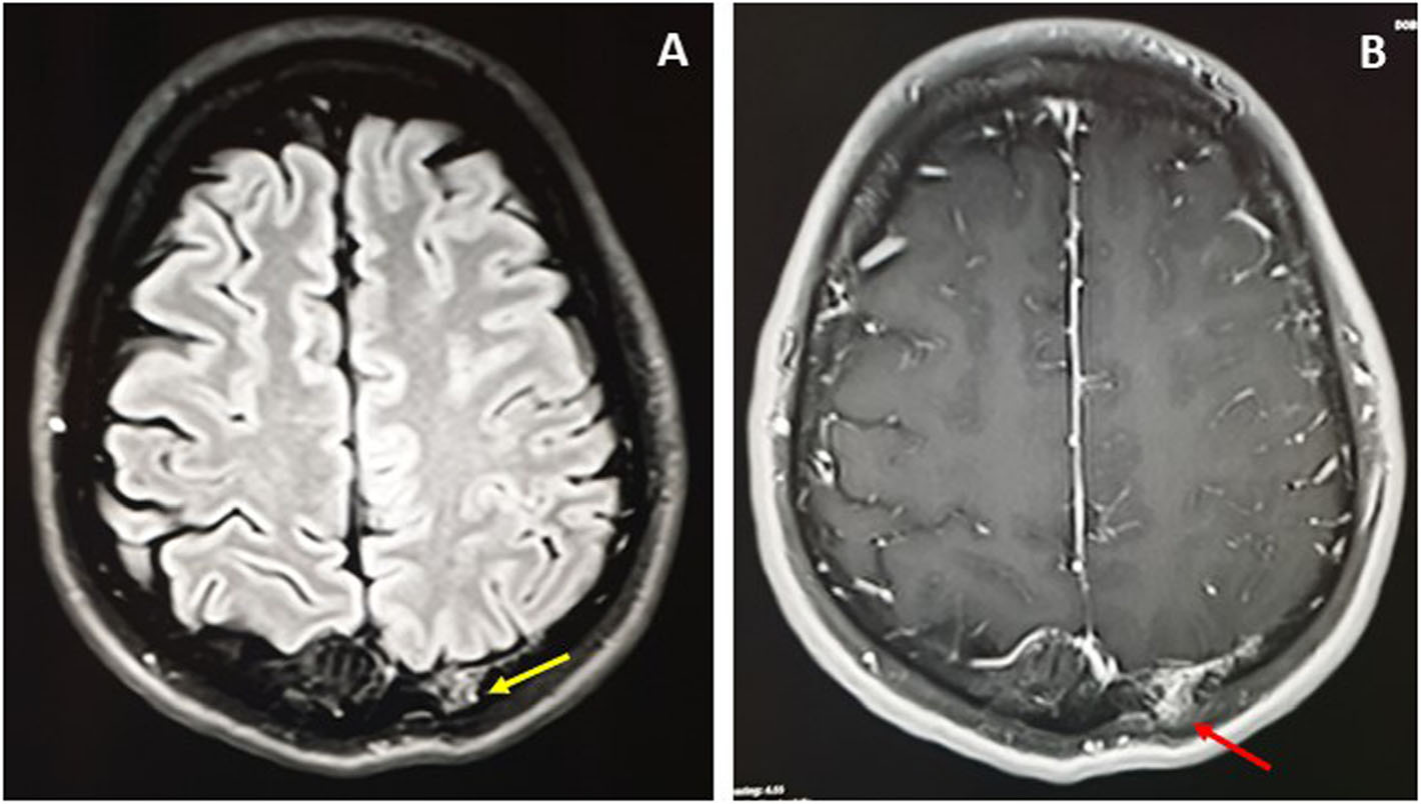
Fluid attenuated inversion recovery (FLAIR) MRI (**a**), and post contrast T1 (**b**) show the left parietal diploic lesion with external wall erosion (Yellow arrow) and subgaleal soft tissue enhancement (Red arrow)

**Fig. 5:**
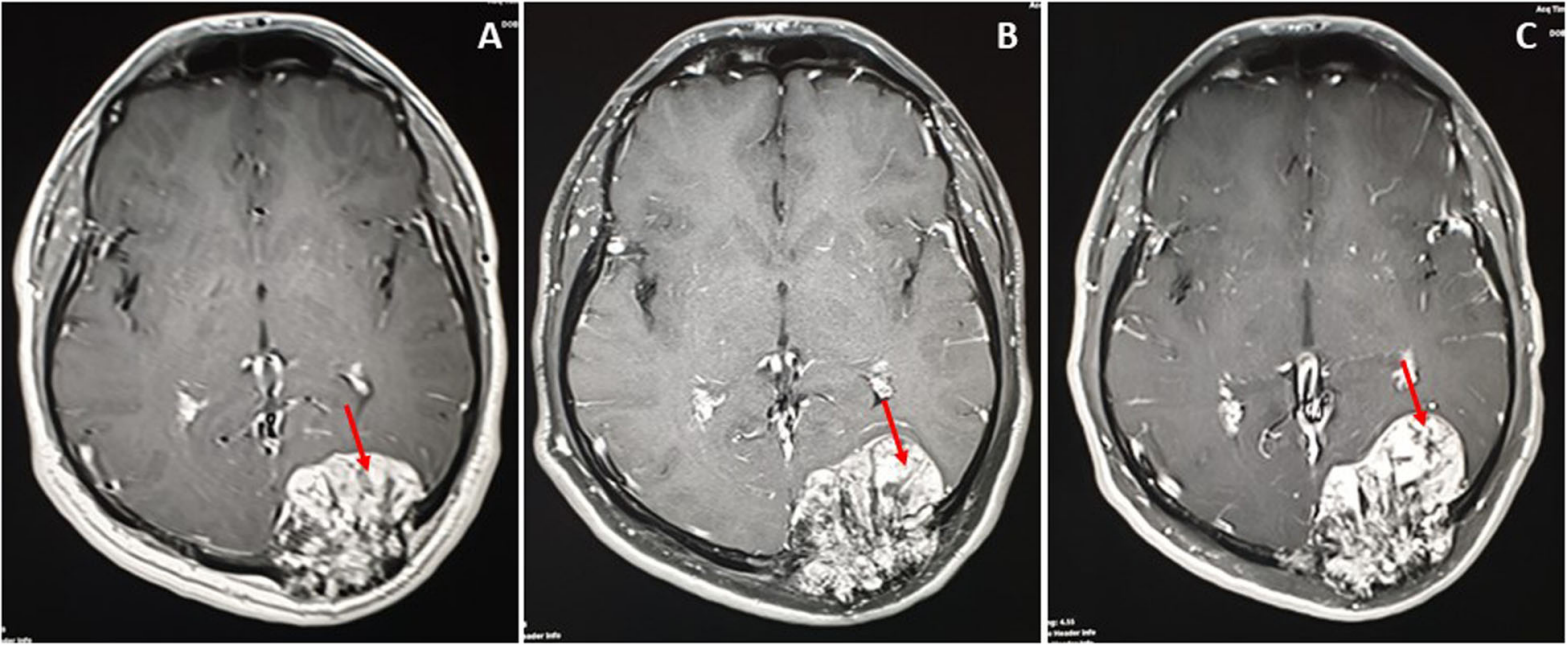
Serial axial MRI (post contrast T1) done on February 2017 (**a**); January 2018 (**b**) and April 2019 (**c**), showing progressive enlargement of the soft tissue component and increased mass effect

**Fig. 6:**
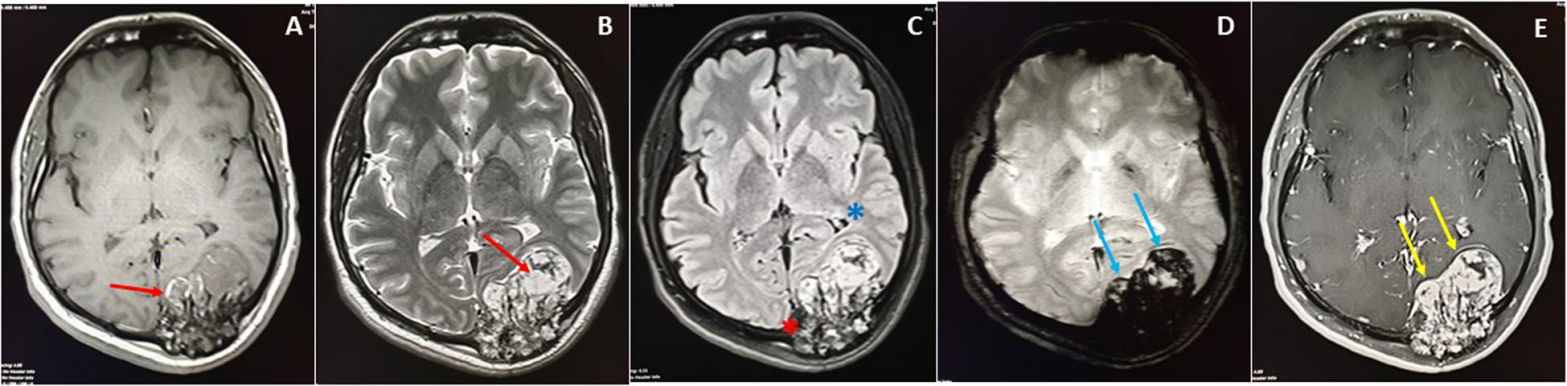
MRI images T1WI (**a**), T2WI (**b**), FLAIR (**c**), Hemo (**d**), and post contrast (**e**) the left occipital lesion showed hyperintensities on T1WI/T2WI of fat and vascular content respectively (Red arrow), and blooming effect (Blue arrows) on Hemo images of hemorrhagic content, and intense post-contrast enhancement (Yellow arrows). It compressed the adjacent occipital lobe without edema, deviating anteriorly the occipital horn (Blue asterisk) of the left lateral ventricle, and obstructing the posterior aspect of the superior sagittal sinus (Red asterisk)

The MRI showed hyperintensities on T1-weighted image (WI) and T2WI of the fat and vascular contents, respectively, and the post-contrast enhancement, without restricted diffusion, was suggestive of calvarial hemangiomas. The left high parietal lesion was compressed and invaded the superior sagittal sinus (Fig.4a). The largest left occipital lesion showed erosion of the skull, with large intracranial enhancing soft tissue mass of the hemorrhagic content (Fig.4b). The serial axial MRI T1 post-contrast images were captured in February 2017, January 2018 and April 2019, and showed progressive enlargement of the soft tissue component and increased mass effect (Fig.5a-c). In Fig.6a-e, the MRI images, T1WI, T2WI, FLAIR, Hemo, and post-contrast are shown; the left occipital lesion showed hyperintensities on T1WI/T2WI of the fat and vascular content, respectively (Fig.6a and b), it also compressed the adjacent occipital lobe without edema, deviated, anteriorly, the occipital horn of the left lateral ventricle, and obstructed the posterior aspect of the superior sagittal sinus (Fig.6c). The blooming effect on the hemorrhagic content was clear from the Hemo images (Fig.6d), and intense post-contrast enhancement (Fig.6e).

**Fig. 7:**
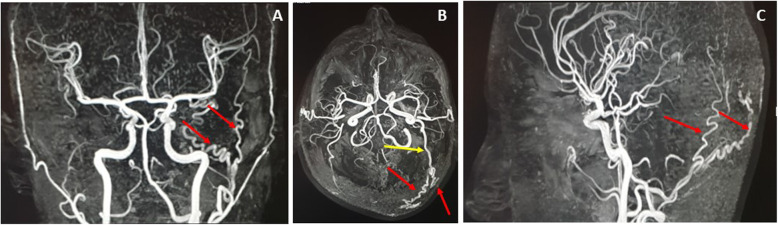
CE-MRA MIP coronal (**a**), axial (**b**), and sagittal (**c**) showed two tortuous arteries (Red arrows) arising from the prominent left occipital artery (Yellow arrow) giving blood supply to the left occipital lesion

**Fig. 8:**
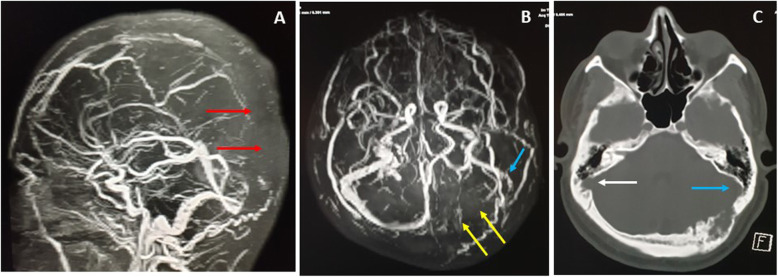
CE-MRV MIP (**a**) sagittal and (**b**) axial showed absent enhancement of the superior sagittal dural sinus posteriorly (Red arrow), and the left transverse dural sinus (Yellow arrows). The left sigmoid dural sinus is small/ hypoplastic (Blue arrow). Panel **c**, is a CT scan bone window showing a shallow left sigmoid groove (Blue arrow) as compared to the right deep groove (White arrow)

From the contrast-enhanced MR angiography, there were two tortuous arteries arising from the prominent left occipital artery (Fig. 7), providing a blood supply to the left occipital lesion. Contrast-enhanced MR venography revealed the absence of flow enhancement at the superior sagittal sinus by the right parasagittal parietal, left occipital lesions and prominent right veins of Labbe and Trolard. In addition, there was absence of flow enhancement at the left transverse dural sinus, with small left sigmoid dural sinus and internal jugular vein was observed (Fig.8a and b). The associated shallow left sigmoid groove, visualized on the CT scan (Fig.8c), may have either indicated chronicity and the result of the long-term effect of the left occipital lesion, or that it was not-related and could be congenital hypoplasia instead.

**Fig. 9:**
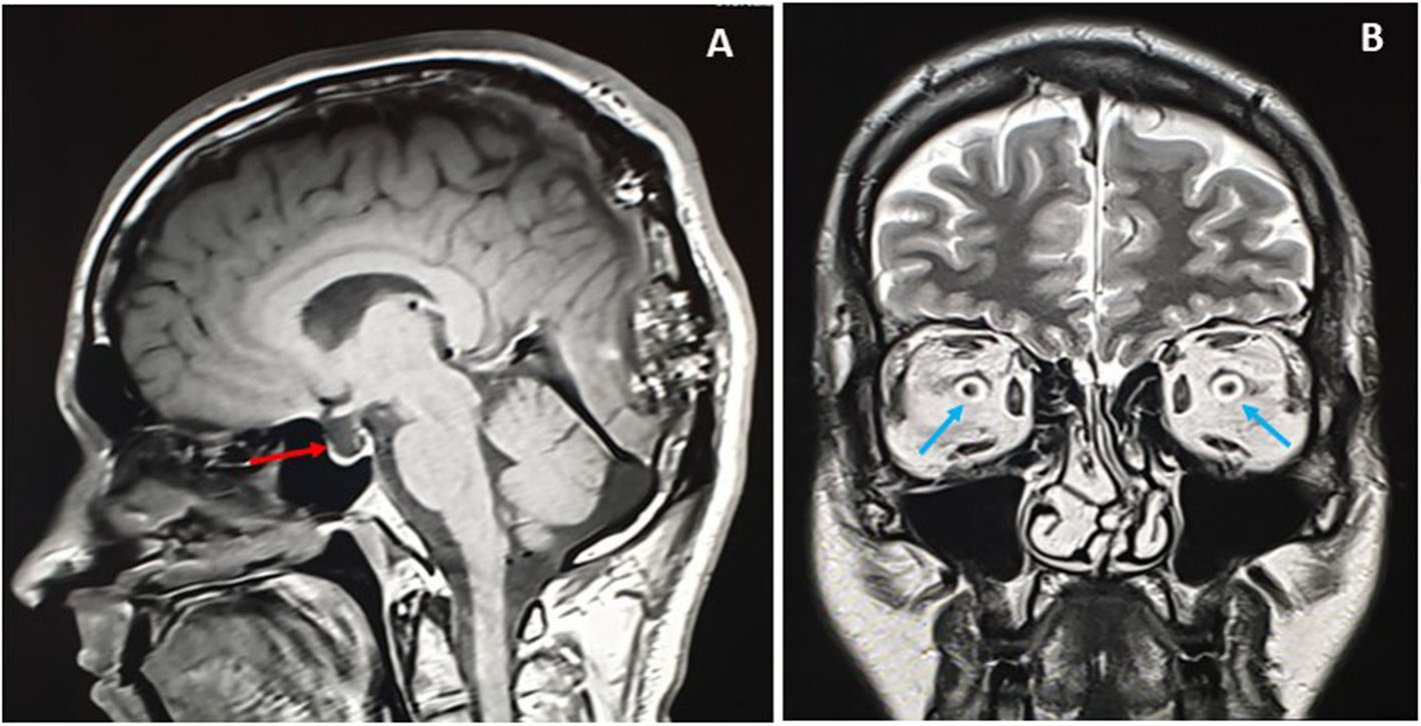
Sagittal T1 MRI image showing empty Sella turcica (red arrow) (**a**) and distended optic nerve sheaths (Blue arrows) on coronal T2 (**b**)

There was also an increased intracranial pressure evident by the empty Sella turcica and distended optic nerve sheaths on the MRI (Fig.9a and b). The neurosurgery team made the differential diagnosis of a diploic dermoid tumor.

### Treatment and prognosis

**Fig. 10:**
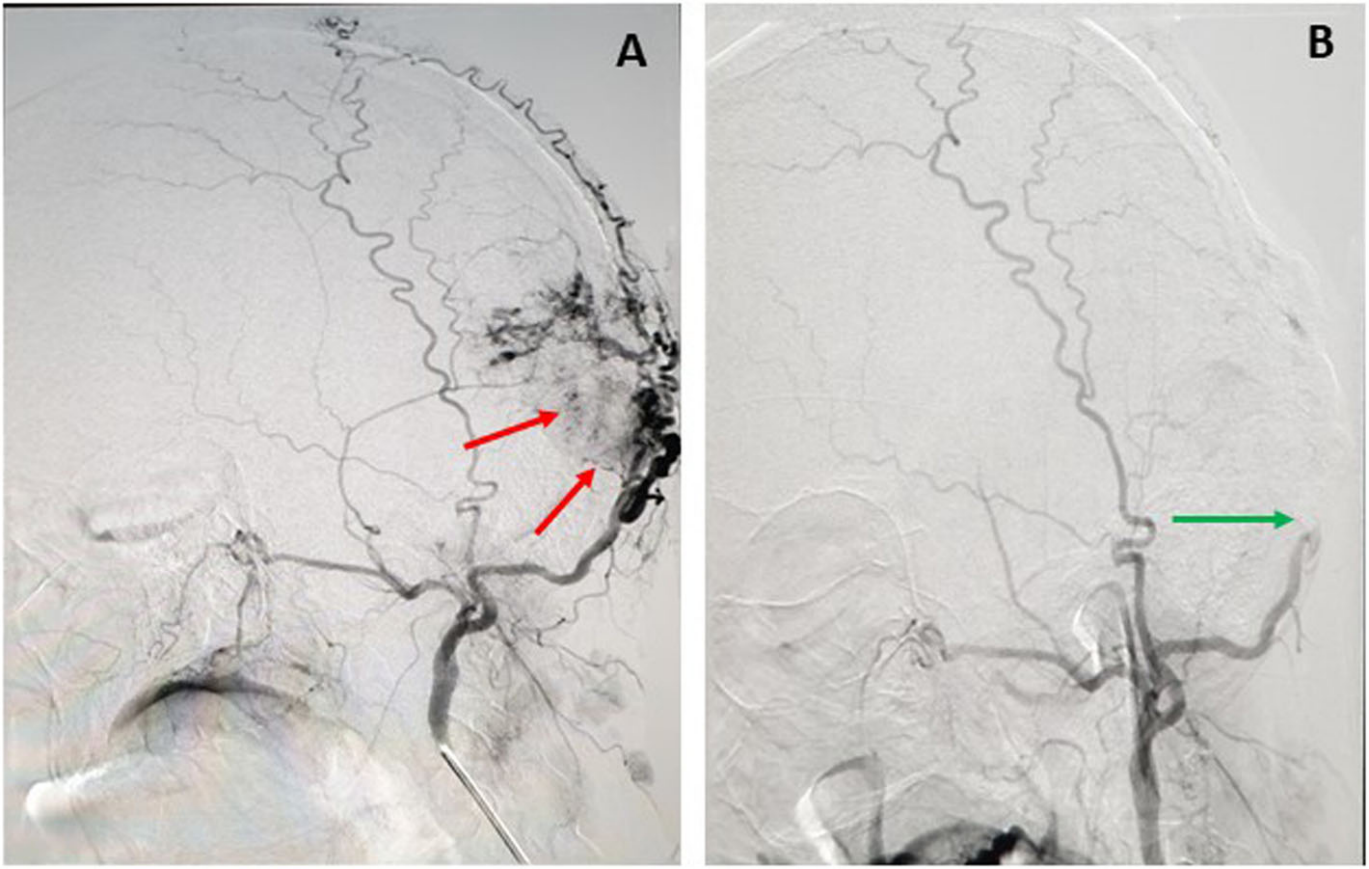
Hypertrophied and tortuous left ECA occipital branch with persistent tumor blush (Red arrows), (**a**). Post embolization of the feeders from the left occipital artery and the left middle meningeal artery (Green arrow), (**b**)

Over the years the patient refused surgery; however, after the last severe presentation, he finally agreed to treatment. Based on the radiological report, cerebral angiogram and embolization of the feeders were performed to minimize the intraoperative bleeding (Fig.10a and b).

**Fig. 11:**
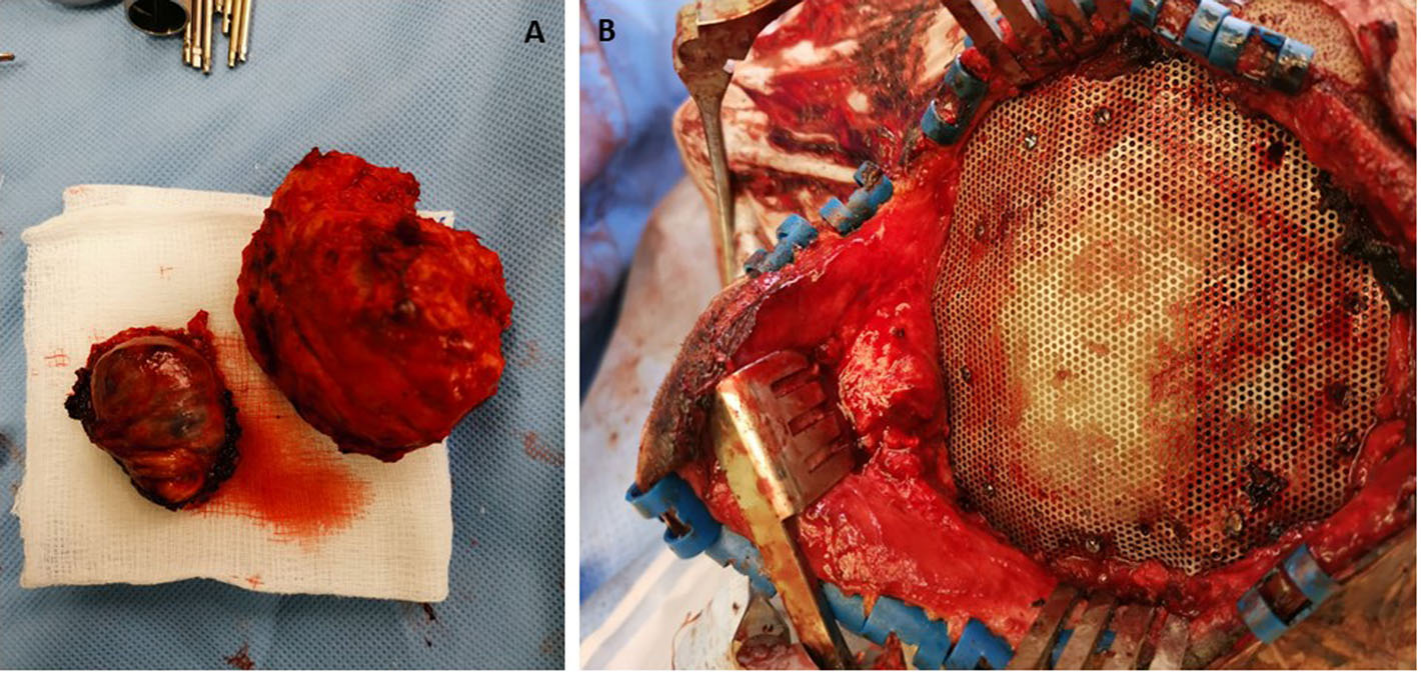
Excised left parietal lesion and largest left occipital lesion (**a**). Bone defect was managed with application of Titanium mesh (**b**)

After 48 h, the patient underwent surgery. Considering that the outer palpable part of the mass was much less than the intracranial extension, it was adjacent to the diploic calcified mass and the bone had thickened, a craniectomy was planned over the adjacent left-sided parietal and occipital mass lesions, under general anesthesia. The head was fixed in the prone position, with the head fixed on a Mayfield head-holder. During the craniectomy, the involved bone was much thicker and was too hard (ivory-like); therefore, the neurosurgeon used numerous tools (a high-speed drill, a bone cutting nozzle of the ultrasonic aspirator, bone nibblers and a Kerrison rongeur) to perform the craniectomy. The part of the mass that was extending intracranially was firm with moderate vascularity and found to be eroding the dura and evaginating the brain tissue. However, there was a clear boundary, with an arachnoidal plane between the tumor and the brain tissue, that facilitated the separation of the tumor with complete excision and ensuring that the brain tissue was intact without violation. The two adjacent lesions were totally excised and hemostasis secured (Fig.11a). Dural grafting to seal the wide dural defect was achieved with the use of an artificial dural substitute (Duraform™). The bone defect was managed by the application of a Titanium mesh, with tiny screws. The scalp was healthy and closed in layers (Fig.11b). The right parietal small intradiploic lesion was outside of the surgical field; therefore, it was left untouched for follow-up.

**Fig. 12:**
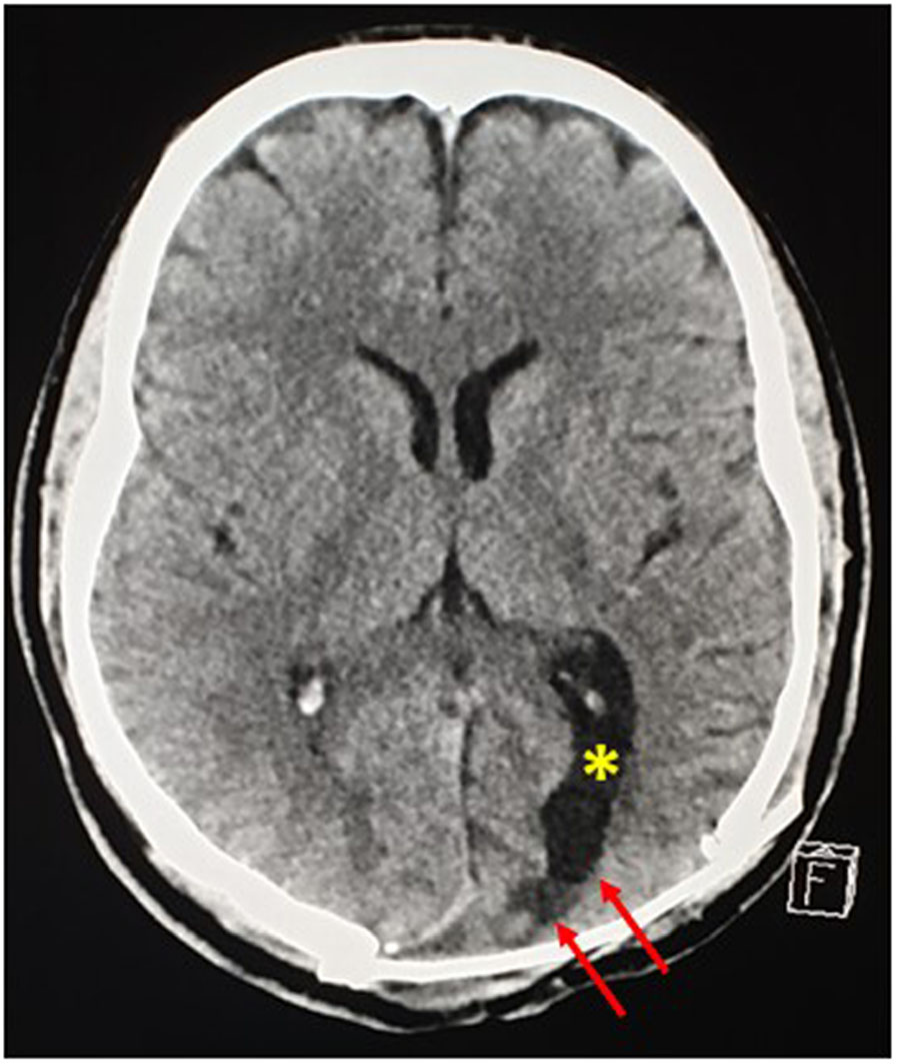
A Computed tomography scan post excision of the left occipital diploic lesion showed atrophic left occipital lobe with encephalomalacia (Red arrows), and ex-vacuo dilatation of the occipital horn of the left lateral ventricle (Yellow asterisk)

Postoperatively, the patient recovered smoothly, was fully conscious, neurologically intact, feed orally and was discharged in a satisfactory condition. Superficial scalp wound infection was observed in the later stages, which was managed by broad-spectrum antibiotics. These wounds were completely healed, and became dry and clean. The patient had a normal life without recurrent symptoms, suggesting a compressive mass effect caused the fits. A follow-up CT brain scan after a few years and months revealed the atrophic left occipital lobe, with encephalomalacia and ex-vacue dilatation of the occipital horn of the left lateral ventricle (Fig.12).

### Histopathology and immunology

**Fig. 13:**
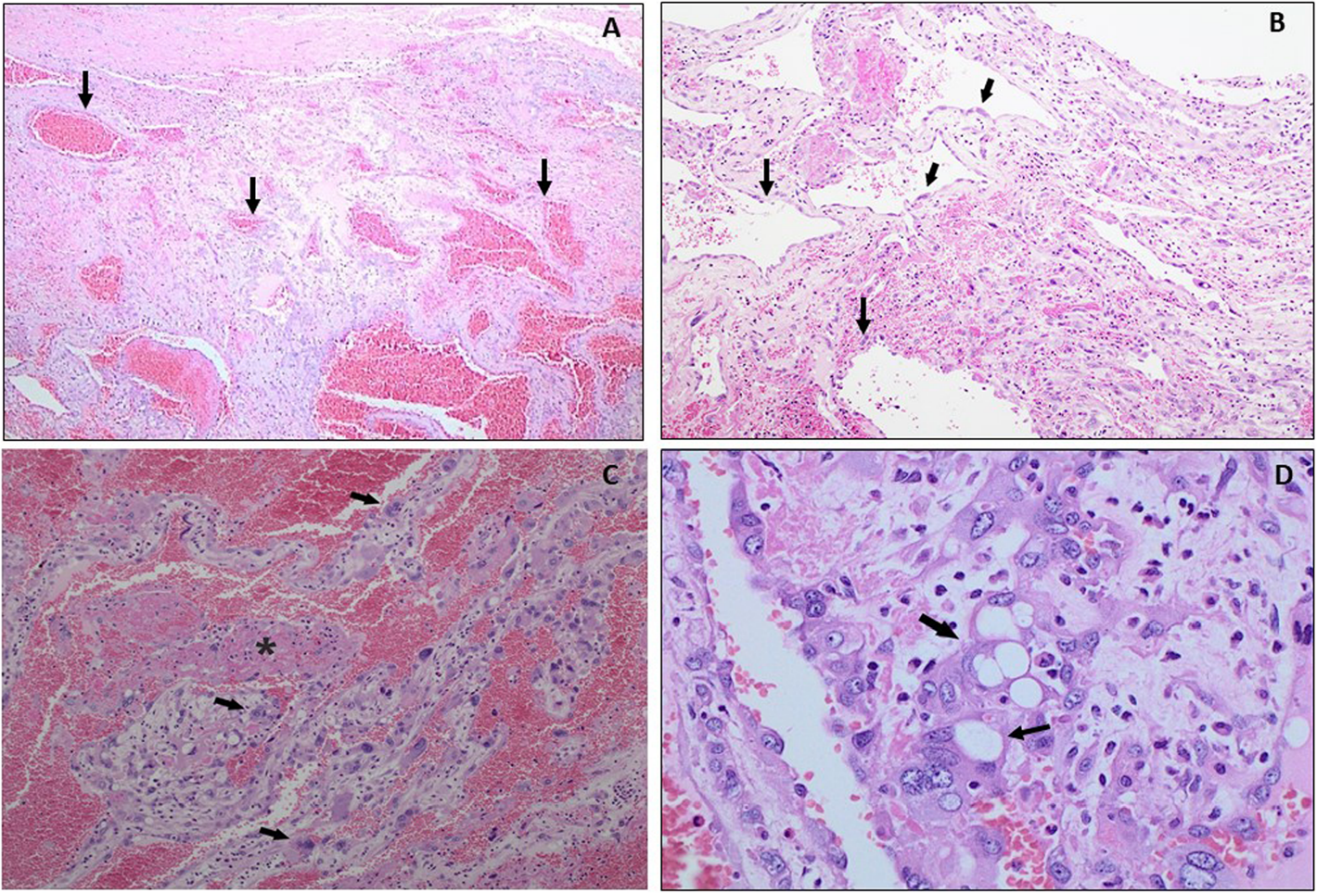
Histopathology of symplastic hemangioma showing, (**a**) Well- defined lesion with variable sized dilated blood vessels (Black arrows), H and E, × 40; (**b**) Vascular endothelial cells with minimal nuclear atypia (Black arrows), H and E, × 100; (**c**) degenerative nuclear atypia, multinucleation (Black arrows) and vascular thrombosis (Black asterisk), H and E, × 200; (**d**) lipoblast-like cells (Black arrow) and scattered inflammatory cells in the background, H and E, × 400

**Fig. 14:**
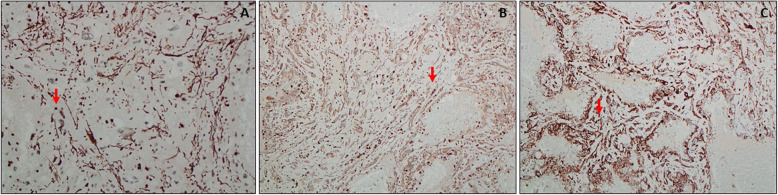
Immunohistochemistry of symplastic hemangioma showing, (**a**) focal positivity for smooth muscle actin (SMA) in pleomorphic cells, × 200; (**b**) CD68 showed weak staining in stromal cells, × 200; (**c**) CD34 positivity in endothelial and stromal cells, × 200

Histopathology of the small left parietal lesion revealed cavernous hemangioma, while the left large occipital lesion showed a vascular lesion, with a thick wall and interstitial cells within a pre-existing vascular lesion. A microscopic examination revealed a well-defined lesion composed of variable sized dilated blood vessels, lined by endothelial cells exhibiting minimal nuclear atypia, with no multilayering or mitosis (Fig.13a and b). Vascular smooth muscle and stromal cells showed degenerative atypia, with enlarged hyperchromatic nuclei and multinucleation (Fig.13c). Lipoblast-like cells and scattered inflammatory cells in the background were also observed (Fig.13d). Focal vascular thrombosis and papillary endothelial hyperplasia were found. The stroma showed hemorrhage, edema and inflammatory cells. Immunohistochemistry data revealed positive staining for smooth muscle actin (SMA) in the pleomorphic cells (Fig.14a), CD68 showed weak staining in the stromal cells (Fig.14b), and CD34 was positively stained in the endothelial cells and some stromal cells (Fig.14c). Ki-67 proliferation index was < 1%.

### Previously published cases of symplastic hemangiomas

In Table 1 we have summarized the previously published cases reports of symplastic hemangiomas and also the immuno-markers used to characterize these tumors. In the past 26 years, only eleven cases of symplastic hemangiomas have been published. The first three (one mediastinal and two cutaneous) cases were described in 1994 [12], another three cutaneous cases were reported in 2000 [13], then in 2006, Goh et al., [10] reported two cutaneous cases; in 2018, a case of intrathoracic symplastic hemangioma [14]; also one case in the nasopharyngeal region in 2015 [15], and a case of infantile (symplastic) hemangioma was reported in 2019 [16]. Our case report of calvarial symplastic hemangioma is the twelfth case report, and the first in the calvarial region.

**Table 1:** Previously published cases of Symplastic Hemangiomas and their Immuno-pathology

Clinical Symptoms	Location	Sex	Age (Years)	Country of Origin	Reference	Immunology
A tumor mass with capillary arteriovenous malformation for 1 year.	Nasopharyngeal	Female	12	Serbia	Samardzija et al.,	The pleomorphic cells in the vascular wall and interstitium were focally positive for SMA and scattered pleomorphic interstitial cells were CD68-positive. SMA, CD31 and CD34 positive in smooth muscle cells and endothelial cells. Endothelial cells were GLUT-1-negative. CD34 in pleomorphic cells was negative. Desmin staining was positive only in the vascular smooth muscle cells. Neither cell was stained with cytokeratin and S-100. Few pleomorphic interstitial cells were positive for CD163.
A polypoid tumor on the right thigh (duration unknown).	Cutaneous	Male	52	United Kingdom	Goh et al.,	Focal SMA positivity in the pleomorphic cells of the vascular walls. CD68 only weakly stained occasional atypical interstitial cells. The pleomorphic perivascular and interstitial stromal cells were negative for CD31, FLI-1, desmin, and S100. The vascular endothelial cells were positive for CD34, CD31, and FLI-1.
A polypoid skin lesion on right side of the nose for 20 years.	Cutaneous	Female	84		Goh et al.,	Focal SMA positivity in the pleomorphic cells of the vascular walls. In addition, focal strong staining for CD34 was seen in some stromal cells, particularly in the interstitial location. The pleomorphic perivascular and interstitial stromal cells were negative for CD31, FLI-1, desmin, and S100. The vascular endothelial cells were positive for CD34, CD31, and FLI-1.
Lenticular slightly protuberant reddish nodules located above the right scapula, for more than 5 years.	Cutaneous	Female	61	Germany	Kutzner et al.,	Only vimentin positive, SMA staining was weak in vessel walls pleomorphic nuclear cells. Cytokeratin, CD68, Factor VIIIa, and S100 were negative in vessel walls, epidermis, and cutaneous nerves.
Angiomatous nodule. on the extensor side of the left forearm for more than 1 decade.	Cutaneous	Male	63		Kutzner et al.,	
Livid, dermatofibroma-like soft tumor over the extensor side of the right knee, existing for many years.	Cutaneous	Male	48		Kutzner et al.,	
A segmental, superficial, infantile hemangioma on the forehead.	Cutaneous	Female	0.5	Chile	Downey et al.,	The pleomorphic vascular cells were focally positive for SMA and negative for desmin. Glut-1 was positive in the endothelial cells of the symplastic and precursor lesion.
A large intrathoracic mass occupying the right middle lobe of the lung (Suspicious of TB).	Intrathoracic	Male	31	Australia	Li et al.,	Stromal cells were negative for ERG, CD31 and CD34. Pleomorphic cells in the vessel walls were SMA positive, atypical stromal cells interstitium were positive for vimentin, and some were positive for SMA and CD68.
1-month history of swelling over the temporal region of the scalp.	Cutaneous	Male	57	Hong Kong, United Kingdom, and USA	Tsang et al.,	The vascular endothelial cells were positive for Factor VIIIa, *Ulex europaeus* lectin, CD31, and CD34.
A long-standing mass in the first web-space of the left hand,	Cutaneous	Female	65		Tsang et al.,	
A large mediastinal mass discovered incidentally.	Mediastinum	Male	55		Tsang et al.,	

## Discussion and conclusions

Calvarial symplastic hemangioma, when compared with calvarial hemangiomas, is rarely reported in the literature. They are benign, slow-growing, malformed vascular lesions, often and incidentally discovered on radiological images obtained for other reasons. They account for < 1% of all primary bone neoplasms. Hemangiomas malignant degeneration has not been reported so far [17, 18]. Histologically, hemangiomas are classified according to the predominant type of vascular channel into four variants, cavernous, capillary, arteriovenous, and venous, and these variants are known to coexist [5, 19]. The cavernous type is predominant in hemangiomas of the skull [20].

The cavernous hemangiomas are characterized by the cluster of dilated blood vessels separated by fibrous septa. This is in contrast to capillary hemangiomas, which consist of small vascular lumens, without many fibrous septa. Cavernous hemangiomas are more common in the skull, whereas the capillary type is more common in the vertebral column [21, 22]. The pathogenesis of calvarial hemangiomas is not yet fully known; however, previous reports suggested that they are congenital [23–25]. The proliferation and differentiation of the undifferentiated primitive mesenchymal cells, induced by the various stimuli-like traumas, may be the potential etiology. Calvarial hemangiomas are common at the frontal bone, followed by the parietal and occipital bone and are rarely reported at the base of the skull [1, 20, 26]. Calvarial hemangiomas usually involve the outer table of the skull, and extensive involvement of the inner table and dura are typically rare [18]. They are usually asymptomatic; however, they may become symptomatic if they are large enough, with a compressing effect [20]. Neurological deficits are uncommon in calvarial hemangiomas, and seizures, as a presenting symptom, are unusual, whereas epileptic seizures are the most frequent symptom in patients with cerebral cavernous malformations [27], and epilepsy was reported in 65% cavernomas cases in the supratentorial region of the brain [28]. Apart from our case, with a history of epilepsy, another case was reported with seizure disorder in calvarial hemangioma [29]. In the present case, and also in the previously reported case, both the inner and outer tables of the skull were involved in causing a compression of the brain [29]. Perhaps, when the tumor grew larger at a later stage, the tumor eroded the meninges and was in direct contact, resulting in the pressure on the brain tissue, which then caused it to behave like a meningioma, with recurrent fits. This may be the reason for the seizure disorder observed in both cases, due to this type of lesion growing slowly and the symptoms appearing after a prolonged period. The case report from Kanu et al., [29] and our case demonstrates that seizure disorder can occur in calvarial hemangiomas, as a rare complication, likely due to a compression effect, as both patients were epilepsy-free after surgery.

From a CT scan, calvarial hemangioma is classically shown as an expansile lytic lesion, with the sclerotic rim of sunburst or a spoke wheel appearance of trabecular thickening radiating from a common center [30]. Our present case showed the classic sunray appearance on tangential radiographs and a typical ‘sunburst pattern’ on CT bone window scans, which is a characteristic of calvarial hemangioma [31]. Erosions of both internal or external plates can occur and may be associated with internal or external tumor expansion. Sometimes, bony trabeculae can grow beyond the cortical bone, and simulate an aggressive pseudo- “hair-on-end” periosteal reaction [32]. From MRI, the signal intensity depends on the fat content, which is hyperintense on T1 and T2. The signal intensity increases with the post-contrast enhancement on CT and MRI. The present case was treated with preoperative embolization, surgical resection, and cranioplasty [33]. Angiography is important for the embolization of feeders for large lesions to avoid intraoperative hemorrhage [34, 35].

As in our case, histopathology showed a symplastic hemangioma, within a pre-existing vascular tumor with minimal endothelial cell atypia, which can be suggested radiologically by the intracranial soft tissue content with hemorrhage. Immunohistochemistry showed focal positivity for SMA in the atypical cells, confirming a symplastic hemangioma, within a preexisting vascular lesion. CD34 antibody staining was performed to confirm the diagnosis and the endothelial cell lining of the vascular channels was found to be positive; thus, confirming our diagnosis. The distinctive lack of endothelial nuclear atypia allowed the distinction from malignant vascular tumors [10, 13–16]. Degenerative atypia of vascular smooth muscle and interstitial cells, mimicking a pseudo malignancy within a preexisting long-standing vascular lesion, with minimal endothelial cells atypia, is a main characteristic of symplastic hemangioma [10, 13]. Immunohistochemical stains showed that many of the pleomorphic cells in the vessel walls were SMA-positive, confirming them to be smooth muscle cells of the vascular media. To the best of our knowledge, this is the first case reported with symplastic hemangioma in the calvarial region.

In a unique case of a male patient, who underwent CT of the chest for a cough and night sweat symptoms, the CT scan showed a large intrathoracic mass in the right middle lobe of the lung, initially raising the suspicion of tuberculosis. This tumor resembled a mediastinal angiosarcoma, with histology features of a highly vascular nature of the lesion, with the focal areas of complex anastomosing vascular channels. However, careful histological and immunological investigations confirmed this case was symplastic hemangioma [14]. Few previous reports of pathological cases masquerading as symplastic hemangiomas are also known. For example, differentiating multinucleate cell angio-histiocytoma (MCAH) from symplastic hemangioma is very difficult, as both of them share common histopathological features, such as the presence of vascular channels, bizarre cells, inflammatory stroma, and multinucleate giant cells mimicking a sarcoma [36]. However, MCAH is more common in extremities, whereas symplastic hemangioma is common on the scalp and face. If the lesion is not present superficially, then there are more chances of misdiagnosis, as reported in the intrathoracic symplastic hemangioma case report [14].

Previously reported cases of skull base calvarial hemangiomas are rare. They occur most frequently in the vertebral column (30–50%) and the skull (20%), whereas the involvement of other sites, such as the long bones, short tubular bones, and ribs is uncommon. Calvarial hemangiomas are more common in females compared with that in males, with a 3:2 ratio, and usually affect individuals 20–40 years of age [20]. In PubMed, 86 reports were found for a “calvarial hemangiomas” search, whereas a search for “symplastic hemangiomas” only revealed 8 reports published until now. The majority of the published symplastic hemangioma cases were of cutaneous origin [10, 12, 13, 16]; one case was intrathoracic in the mediastinum [14], another one was in the nasopharyngeal mucosa [15], and Xu et al., [37] have reported a symplastic hemangioma case from China the location of this lesion is not known. The reported cases were also in middle-aged to the elderly, except one case of symplastic hemangioma, which was a pediatric infantile hemangioma arising over a skin lesion in a 6-month-old child [16], and the second pediatric case, of symplastic hemangioma was in the nasopharynx region arising extracutaneously over a capillary arteriovenous malformation [15]. A rare case of symplastic trichodiscoma was also reported in a 50-year-old male, who presented with a nodule on the nose for 6 years. This case was reported to have histological features similar to ancient schwannoma and symplastic hemangioma [38]. Trichodiscoma is a type of neurofollicular hamartoma, which is an abnormal malformation of the skin [39]. The term “ancient schwannoma” is used to describe a schwannoma, with degenerative nuclear changes, that may be misinterpreted as a sarcomatous change [40].

Other large intra-diploic skull lesions to be considered in differential diagnosis of calvarial hemangioma include osteosarcoma, giant cell tumor (GCT), epidermoid cyst, and aneurysmal bone cyst (ABC). Although osteosarcomas are rarely affecting the skull, but they show sunburst appearance, and specular calcifications on CT Scan [41]. The GCTs show marginal sclerosis and solid-cystic components indicating remodeling and destruction. On MRI, they show mixed density in both T1 and T2W images, and post contrast heterogenous enhancement [42]. The intra-diploic epidermoid cysts are expansile lytic bony lesions with well-defined margins, and hypodense on CT scan [43, 44]. Radiographically, epidermoid cysts erode expanding into inner and outer tables of the calvaria. They show characteristic high signal on DWI MRI with no or minimal peripheral post contrast enhancement and no enhancement within the cyst [45]. They are filled with debris (keratin and cholesterol) from the desquamation of their thin squamous epithelial lining, usually the cyst has a pearl-like appearance [46]. The ABCs are commonly seen in younger age group, usually before the age of 20 years with characteristic “soap bubble-like” appearance for the multi-cystic mass on CT Scan with thinning of bony cortex with infrequent new bone formation around the lesions. On MRI, these lesions manifest with fluid-fluid levels and peripheral rim enhancement [47].

Overall, our case report has addressed calvarial symplastic hemangioma, which when compared to calvarial hemangiomas, is rarely reported in the literature. This has similar imaging features to calvarial hemangiomas, which classically show expansile lytic lesions with the sclerotic rim of sunburst or a spoke-wheel appearance on a CT scan. On MRI, the signal intensity depends on the fat, soft tissue and hemorrhagic content, and shows post-contrast enhancement. As in our case, a symplastic hemangioma can be suggested by a slow-growing soft tissue mass with hemorrhage in a pre-existing hemangioma. Symplastic hemangioma is a histopathological diagnosis, but radiology also confirmed this in our case. MRI was performed consecutively for 3 years, which showed progressive enlargement of the soft tissue component and increased mass effect in a pre-existing hemangioma.

## Data Availability

The datasets used and/or analyzed during the current study available from the corresponding author on reasonable request.

## Abbreviations

ABC: Aneurysmal bone cyst
CE-MRA: Contrast-enhanced MR angiography
CE-MRV: Contrast-enhanced magnetic resonance venography
CT: Computed tomography
DWI: Diffusion-weighted imaging
ECA: External carotid artery
FFPE: Formalin fixed paraffin embedded
FLAIR: Fluid attenuated inversion recovery
GCS: Glasgow coma scale
GCT: Giant cell tumor
H and E: Hematoxylin and eosin stain
IJV: Internal jugular vein
MCAH: Multinucleate cell angio-histiocytoma
MIP: Maximum intensity projection
MRI: Magnetic resonance imaging
NECT: Non-enhanced computed tomography
SMA: Smooth muscle actin
